# Geographic variations and temporal trends of *Salmonella*-associated hospitalization in the U.S. elderly, 1991-2004: A time series analysis of the impact of HACCP regulation

**DOI:** 10.1186/1471-2458-9-447

**Published:** 2009-12-03

**Authors:** Kenneth KH Chui, Patrick Webb, Robert M Russell, Elena N Naumova

**Affiliations:** 1 Department of Public Health and Family Medicine, Tufts University School of Medicine, Boston, MA, USA; 2 Friedman School of Nutrition Science and Policy, Tufts University, Boston, MA, USA; 3 Jean Mayer USDA HNRCA, Tufts University, Boston, MA, USA

## Abstract

**Background:**

About 1.4 million *Salmonella *infections, a common food-borne illness, occur in the U.S. annually; the elderly (aged 65 or above) are most susceptible. In 1997, the USDA introduced the Pathogen Reduction and Hazard Analysis and Critical Control Points Systems (PR/HACCP) which demands regular *Salmonella *testing in various establishments processing meat products, such as broiler chickens. Impact evaluations of PR/HACCP on hospitalizations related to *Salmonella *are lacking.

**Methods:**

Hospitalization records of the U.S. elderly in 1991-2004 were obtained from the Centers of Medicare and Medicaid Services. Harmonic regression analyses were performed to evaluate the long-term trends of *Salmonella*-related hospitalizations in pre- and post-HACCP periods. Seasonal characteristics of the outcome in the nine Census divisions of the contiguous U.S. were also derived and contrasted.

**Results:**

Predicted rates decreased in most divisions after 1997, except South Atlantic, East South Central, and West South Central. These three divisions also demonstrated higher overall hospitalization rates, pronounced seasonal patterns, and consistent times to peak at about 32^nd ^to 34^th ^week of the year.

**Conclusion:**

The impact of HACCP was geographically different. South Atlantic, East South Central, and West South Central divisions should be targeted in further *Salmonella *preventive programs. Further research is needed to identify the best program type and timing of implementation.

## Background

*Salmonella *infection is a common food- and water-borne disease in the U.S.; about 1.4 million cases occur annually [1], of which 22% lead to hospitalization. Infants, children, and elderly are particularly at risk [2,3]. For infected elderly (65 year-old or above), the chance of hospitalization rises to 47%-80%. The case-fatality rate due to *Salmonella *infection is also highest among elderly (3.5%, compared to the average 0.6%) [4,5]. As the U.S. is rapidly aging, preventing *Salmonella *infections in the elderly is important [6,7].

In addition to compromised life quality due to the symptoms induced by the illness, *Salmonella *infections are also expensive to treat. Between 1990 and 1999, the mean cost of a *Salmonella*-associated hospitalization was about US$ 7,400 [3]. The total estimated medical cost in 2006 due to *Salmonella *infections was US$191 million [8]. This medical expense is very likely to rise with the expanding proportion and size of the elderly population.

On 25^th ^July 1996, the Food Safety and Inspection Service (FSIS) of the U.S. Department of Agriculture (USDA) declared its final rule on Pathogen Reduction and Hazard Analysis and Critical Control Points (PR/HACCP) Systems. This rule demanded regular tests for *E. coli *in selected food industries. Mandatory testing for *Salmonella *was also introduced in all meat and poultry establishments, depending on their size, between 1997 and 2000. A pre-post study showed a reduction in *Salmonella *prevalence in broiler chickens, swine, and ground beef [9].

The long term results of the implementation of the HACCP System have been mixed. Overall, there has been a decrease in *Salmonella *infections. However, when segregated into different serotypes, only serotype Typhimurium decreased significantly, the more common serotype Enteritidis actually increased [10]. An increasing trend of positive test results for Enteritidis was also documented in broiler chickens from 2000 to 2005 [11]. No study investigating the long-term association between HACCP and *Salmonella*-associated hospitalizations in elderly has been identified.

*Salmonella *infection rates had been decreasing from 1987 through 1997 in most parts of the United States [2]. The U.S. Healthy People 2010, a set of health objectives designed to identify the most significant preventable threats to health and to establish national goals to reduce these threats, suggested the further reduction of *Salmonella *infections from 13.7 cases to 6.8 cases per 100,000 people from 1997 to 2010 [12]. However, according to the CDC, the number of *Salmonella *infections had actually increased to 14.7 cases per 100,000 people in 2004 [13]. Therefore reaching the objective proposed by the Healthy People 2010 is clearly going to be challenging.

One potential solution would be to better locate the time and place for implementing preventions. *Salmonella *infection is seasonal: it shows a systematic, repetitive, periodic fluctuation over time [14,15]. Understanding this temporal pattern has allowed better prediction of upcoming epidemics and clarified possible implication of other covariates, such as temperature [16]. Consequently, the efficiency of preventive strategies can be improved through focusing effort at the right time.

Our first objective was to examine the 14-year long-term trend of hospitalizations due to laboratory-confirmed *Salmonella *infection in the U.S. elderly population from 1991 to 2004, by referring to a nationally and systematically collected dataset. We hypothesized that, after adjusting for annual seasonality, there was a substantial decrease in hospitalizations related to *Salmonella *infection in the time duration, and the rate of decrease in such hospitalizations would be higher after 1997, when the HACCP targeting *Salmonella *was introduced. Our second objective was to quantify the annual temporal fluctuation of *Salmonella *infections with harmonic regressions, followed by statistical comparisons of curve parameters such as time to peak, minimum rate, and maximum rate in the nine Census divisions. The results would assist policy makers in deciding the location and timing of further prevention programs tackling *Salmonella *infections.

## Methods

### Data source and preparation

Individual hospitalization records were obtained from the Centers of Medicare and Medicaid Services (CMS). The dataset contained age, residential state and zip code, date of admission, and up to 10 International Classification of Diseases, Ninth Revision, Clinical Modification (ICD-9-CM) diagnostic codes of all the hospitalized Medicare recipients aged 65 or above in the U.S. from 1991 through 2004. The CMS data are nationally representative because Medicare covers about 95% of the U.S. elderly. In addition, hospitalizations due to *Salmonella *infections in the U.S. were shown to be significantly correlated with the surveillance records [3], hence are valid estimations of overall salmonellosis rates.

*Salmonella *infections are represented by two ICD codes which displayed very different incidences: In the 14-year duration, there were only 584 cases of ICD 002, typhoid and paratyphoid fever, but 28,959 cases of ICD 003, other *Salmonella *infections. So, in this analysis, we adopted ICD 003 as the only condition and defined a hospitalization as *Salmonella*-associated if the first three digits of any one of the 10 diagnostic codes were "003". Under category 003 were different sub-codes representing various manifestations of the disease. For instance: 003.0 is gastroenteritis, 003.1 is septicaemia, and 003.21 is meningitis. Serotypes, however, were unavailable in this dataset.

Data aggregations were performed prior to analysis. The individual records were aggregated from daily level (5,114 days) into weekly level (734 weeks), as well as from the county level into Census divisions, which is a geographic categorization used by the Census Bureau that divides the U.S. into nine areas; New England, Middle Atlantic, East North Central, West North Central, South Atlantic, East South Central, West South Central, Mountain, and Pacific [17]. Eight of the Census divisions are composed of contiguous states. The exception, Pacific, includes the states Hawaii (HI) and Alaska (AK). Since *Salmonella *infection is associated with environmental factors such as temperature [15,18], we decided to exclude Hawaii and Alaska from this analysis to maintain the homogeneity of the potential exposures in the Pacific division.

The major outcome was the hospitalizations rate, derived using the population data from Census 1990 and 2000. Weekly divisional elderly populations were estimated with linear interpolation.

This analytical protocol was approved by the Institutional Review Board at Tufts Medical Center/Tufts University Health Sciences Campus.

### Analyzing the change of long-term trends

A linear spline model (also known as broken stick model) was used to discern if the slope changed significantly before and after the start of 1997--when the first stage of the HACCP system on bacterial testing in broilers started.

Long-term trends and accompanying seasonality were assessed with the seasonal-trend decomposition procedure based on LOESS [19], which assumes that a seasonal pattern is an additive result from three components: trend, seasonality, and remainder. Based on this idea, we modified and applied a Poisson regression model to detect the changes of long term trends before and after HACCP introduction while controlling for seasonal fluctuation:(1)

where *Y*_*t *_is a time series of the weekly hospitalization rates modelled with Poisson distribution. The linear terms, *t*_<1997 _and *t*_≥ 1997 _represent the time (in weeks) before and after the HACCP implementation (beginning of 1997), they were constructed so that:

allowing the long term trend of hospitalizations in the pre- and post-HACCP period regression line to inflect at the first week of 1997. The intercept, *β*_0_, represents the predicted outcome at the inflection point. Through comparing the confidence intervals of the coefficients *β*_1 _and *β*_2_, significant change in long-term trend before and after the HACCP implementation can be inferred.

The two trigonometric functions--sin(2*πωt*) and cos(2*πωt*)--serve to adjust for annual oscillations of the outcome; *ω *is the frequency of an annual oscillation expressed in weeks and the constant 2*π *specifies a one-cycle annual oscillation.

### Analyzing the seasonality of the hospitalization outcomes

In order to test whether the annual seasonal characteristics were different, we applied harmonic regression on every single year of the data, from 1991 through 2004, to generate a set of comparable estimates. The model is as follows,(2)

where *k *represents each individual year and other notations are identical to that of formula (1). The estimates *β*_1 _and *β*_2 _in Formula (2) were used to derive the time to peak, minimum, maximum, and relative intensity (also known as peak-to-trough ratio, derived by dividing the maximum by the minimum). Details on this method were presented by Naumova and McNeill [20].

A generalized linear model with Poisson distribution and log link function was used in both linear spline and annual harmonic regression analyses. All data analyses were carried out with S-Plus 8 [21]. Statistical significance is declared based on α = 0.05. Mapping was performed with ArcGIS 2.0 [22]. All geographic analyses were performed to each Census division as well as the whole contiguous United States in order to allow comparisons against the average.

## Results

### Long term trends in the divisional hospitalizations

**Figure 1:**
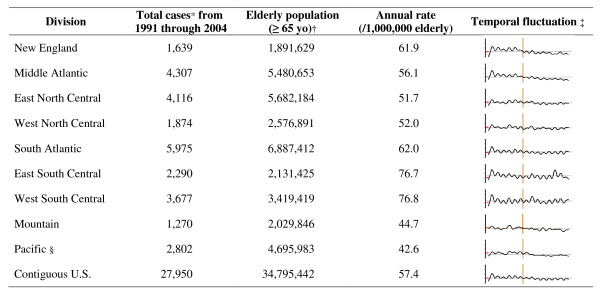
**Descriptive statistics of hospitalization related to *Salmonella *infection in U.S. elderly, 1991--2004**. * Hospitalization records with at least one ICD diagnosis code from the "003" category, which indicates "other *Salmonella *infections". † Based on Census 2000. ‡ Loess curve plotted on uniform scale (span = 7%). Red dash on the left indicates the average of the contiguous U.S. Vertical line at the middles indicates the start of 1997. §Excluding HI & AK.

From about 200 million hospitalization records, we abstracted 27,790 records that involved *Salmonella *infection. Figure 1 shows the hospitalization counts in the 14-year duration, annual rates, and temporal fluctuation represented by sparkline (Loess curve, 5% span) [23] of hospitalizations related to *Salmonella *infection in the contiguous U.S. between 1991 through 2004.

The South Atlantic, East South Central, and West South Central divisions showed pronounced annual oscillations. Long-term decreases were observed in the New England, Middle Atlantic, Pacific, and Mountain divisions.

### Change of hospitalization rates before and after the HACCP implementation

**Figure 2:**
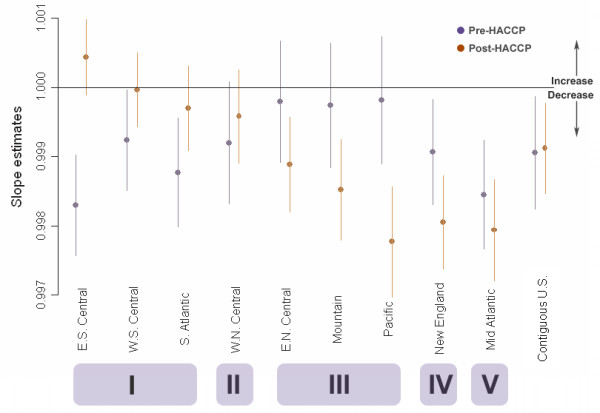
**The change of hospitalization rates before and after the HACCP implementation**. Estimated rates of change of slope of pre- and post- Hazard Analysis and Critical Control Points Systems (HACCP) periods for each Census division derived from the 14-year analysis of a single time series are displayed with corresponding 95% confidence intervals. The outcome is weekly hospitalization rate in cases per 1,000,000 projected elderly, modelled using generalized linear models with Poisson distribution and controlled for annual oscillation. Points falling below (above) the horizontal line indicate a decrease (increase) of rates of change. The pattern of changes can be categorized into five groups: I: significantly decreasing in pre-, stable in post-HACCP; II: Stable in both periods; III: stable in pre-, significantly decreasing in post-HACCP; IV: significantly decreasing in both periods, with the post-HACCP decrease being of significantly greater magnitude; and V: significantly decreasing in both periods.

Table 1 shows the results of the linear spline analysis. The results were rearranged and displayed in Figure 2, in which divisions with similar behaviors in their change of hospitalization rates were grouped and described.

**Table 1:** Results of the linear spline analysis on the change of hospitalization rates due to *Salmonella *infection before and after HACCP* by the nine Census divisions

Division	Intercept	95% CI	β_1_	95% CI	β_2_	95% CI	p-value for slope change
New England	0.27	(0.14, 0.40)	**-0.0009**	**(-0.0017, -0.0002)**	**-0.0020**	**(-0.0026, -0.0013)**	0.12
Middle Atlantic	0.12	(-0.02, 0.26)	**-0.0015**	**(-0.0023, -0.0008)**	**-0.0021**	**(-0.0028, -0.0013)**	0.46
East North Central	0.08	(-0.06, 0.22)	-0.0002	(-0.0011, 0.0007)	**-0.0011**	**(-0.0018, -0.0004)**	0.20
West North Central	-0.04	(-0.19, 0.1)	-0.0008	(-0.0017, 0.0001)	-0.0004	(-0.0011, 0.0003)	0.59
South Atlantic	0.10	(-0.03, 0.23)	**-0.0012**	**(-0.0020, -0.0004)**	-0.0003	(-0.0009, 0.0003)	0.15
East South Central	0.15	(0.03, 0.28)	**-0.0017**	**(-0.0024, -0.001)**	0.0004	(-0.0001, 0.0010)	<0.001
West South Central	0.27	(0.15, 0.39)	**-0.0008**	**(-0.0015, 0)**	0	(-0.0006, 0.0005)	0.21
Mountain	0.04	(-0.10, 0.19)	-0.0003	(-0.0012, 0.0006)	**-0.0015**	**(-0.0022, -0.0007)**	0.10
Pacific†	0.03	(-0.12, 0.18)	-0.0002	(-0.0011, 0.0007)	**-0.0022**	**(-0.0030, -0.0014)**	0.01
Contiguous U.S.	0.10	(-0.03, 0.24)	**-0.0009**	**(-0.0018, -0.0001)**	**-0.0009**	**(-0.0015, -0.0002)**	0.92

Outcome: weekly hospitalization rate (cases per 1,000,000) modelled with Poisson distribution

Predictors: time before HACCP (*β*_1_), time after HACCP (*β*_2_), controlled for annual oscillation

Regression model: log [*Y*_*t*_] = *β*_0 _+ *β*_1_(*t*_<1997_) + *β*_2 _(*t*_ ≥ 1997_) + *β*_3_sin(2*πωt*) + *β*_4 _cos(2*πωt*) + *ε*_*t*_

* Hazard Analysis and Critical Control Points Systems

† Excluding HI & AK

Before the HACCP implementation, New England, Middle Atlantic, South Atlantic, East South Central, and West South Central had been experiencing significant decreases in *Salmonella*-associated hospitalization (p < 0.05); after HACCP implementation, the rate in New England and Middle Atlantic continued to drop, and East North Central, Mountain, and Pacific divisions also showed a significant decrease (p < 0.05).

### Seasonality of the divisional hospitalization rates

**Figure 3:**
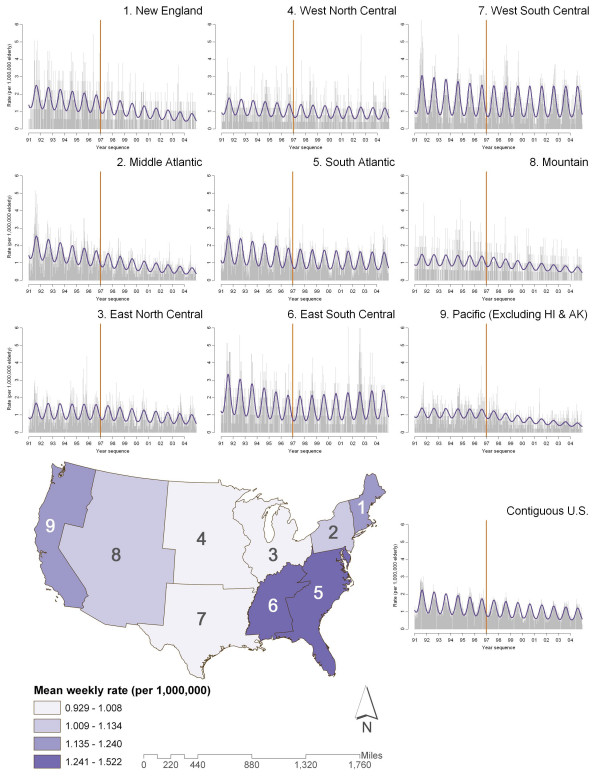
**Seasonality of the national and divisional salmonellosis-associated hospitalization rates**. The time series of weekly rate (cases per 1,000,000 elderly) against year for each of the nine Census divisions was superimposed with predicted values obtained from the annual harmonic regression. The time label of the x-axis was transformed into year for easier interpretation of the annual pattern. The vertical reference line indicates the start of year 1997, the introduction of the Hazard Analysis and Critical Control Points Systems regulation on testing *Salmonella *in major chicken broilers.

Figure 3 displays the time series plots, on which the fitted values of the long-term trend analysis were plotted, for all nine divisions as well as the contiguous U.S. The national map at the center provides average weekly rate information from 1991 to 2004. The vertical reference line indicates the beginning of 1997.

### Selecting the best division and time for preventive interventions

To demonstrate the annual variation, unadjusted between-year confidence intervals were computed (N = 14 years) and displayed in Table 2 for the time to peak, minimum and maximum rates, as well as relative intensity (peak-to-trough ratio). The linear term for the week series (*t*_*k*_) was found to be statistically insignificant in all the models and hence we removed this term from the model. The divisions are ranked by descending order of their annual rates retrievable from Figure 1.

**Table 2:** Seasonal characteristics derived from the annual harmonic regression with between-year confidence intervals

Division*	Periods	Time to peak in week		**Min rate**†		**Max rate**†		Relative intensity (Max rate/Min rate)	
		
		mean	95% CI	mean	95% CI	mean	95% CI	mean	95% CI
West South Central	Pre-HACCP	33.0	(31.9, 34.1)	0.6	(0.5, 0.7)	1.3	(1.1, 1.4)	2.3	(1.9, 2.6)
	Post-HACCP	29.8	(25.5, 34.2)	0.5	(0.5, 0.6)	1.3	(1.2, 1.5)	2.5	(2.2, 2.8)

East South Central	Pre-HACCP	32.8	(30.8, 34.7)	0.6	(0.5, 0.7)	1.2	(1.0, 1.4)	2.1	(1.8, 2.5)
	Post-HACCP	33.3	(31.2, 35.3)	0.5	(0.4, 0.7)	1.1	(0.9, 1.2)	2.1	(1.8, 2.4)

South Atlantic	Pre-HACCP	30.5	(26.4, 34.6)	0.7	(0.7, 0.8)	1.6	(1.4, 1.7)	2.2	(1.8, 2.5)
	Post-HACCP	33.1	(32.3, 34.0)	0.9	(0.8, 0.9)	2.0	(1.8, 2.3)	2.4	(2.0, 2.7)

New England	Pre-HACCP	32.3	(29.3, 35.3)	1.1	(0.9, 1.2)	2.4	(2.1, 2.7)	2.4	(1.7, 3.2)
	Post-HACCP	34.8	(33.7, 35.9)	1.0	(0.9, 1.0)	2.2	(1.8, 2.6)	2.2	(1.7, 2.7)

**Contiguous U.S**.	**Pre-HACCP**	**32.3**	**(29.3, 35.3)**	**0.5**	**(0.4, 0.6)**	**0.8**	**(0.7, 0.9)**	**1.8**	**(1.3, 2.3)**
	**Post-HACCP**	**33.1**	**(32.6, 33.5)**	**0.6**	**(0.5, 0.7)**	**1.4**	**(1.3, 1.5)**	**2.4**	**(2.2, 2.5)**

Middle Atlantic	Pre-HACCP	32.2	(29.8, 34.6)	0.8	(0.7, 0.8)	1.7	(1.5, 1.9)	2.4	(1.9, 2.8)
	Post-HACCP	34.8	(33.1, 36.5)	0.7	(0.6, 0.9)	1.7	(1.4, 1.9)	2.5	(1.8, 3.1)

West North Central	Pre-HACCP	32.4	(31.6, 33.3)	0.7	(0.6, 0.8)	2.9	(2.5, 3.3)	4.2	(3.6, 4.9)
	Post-HACCP	29.4	(24.4, 34.4)	0.8	(0.7, 0.9)	1.5	(1.3, 1.7)	1.9	(1.7, 2.1)

East North Central	Pre-HACCP	33.0	(31.8, 34.2)	0.8	(0.8, 0.9)	2.2	(1.9, 2.5)	2.7	(2.3, 3.1)
	Post-HACCP	30.8	(29.6, 32.0)	0.9	(0.8, 1.0)	2.6	(2.2, 3.0)	2.9	(2.5, 3.2)

Mountain	Pre-HACCP	34.0	(33.5, 34.5)	0.6	(0.6, 0.7)	1.7	(1.6, 1.8)	2.8	(2.3, 3.3)
	Post-HACCP	31.8	(30.5, 33.0)	0.7	(0.6, 0.8)	2.4	(2.0, 2.9)	3.8	(2.8, 4.8)

Pacific‡	Pre-HACCP	33.6	(32.8, 34.4)	0.8	(0.7, 0.8)	2.4	(2.1, 2.7)	3.3	(2.9, 3.7)
	Post-HACCP	28.4	(24.4, 32.5)	0.5	(0.4, 0.6)	1.2	(1.0, 1.3)	2.8	(2.0, 3.5)

* National (in bold) and divisional data are ranked in descending order of annual rates of hospitalization (see Figure 1).

† Weekly, cases per 1,000,000 elderly population

‡ Excluding HI & AK

The peak times of hospitalizations due to *Salmonella *infections ranged between 28^th ^to 35^th ^week of a year (about July till mid August). West South Central, East South Central, and South Atlantic had annual rates exceeding the national reference. They also had significant decrease before HACCP, but such trend became insignificant afterwards (Table 1).

## Discussion

### Is the HACCP regulation associated with lower salmonellosis rates?

It is important to realize that the raw difference in divisional rates is not the outcome of interest for two reasons: (i) the rate of hospitalization is not just a function of disease prevalence but also of other factors, such as the number of hospital beds per citizen, practices of disease coding, and health-seeking behaviors, (ii) the implementation of the HACCP regulation studied here was very unlikely to have impacted on the divisional medical infrastructure, in this analysis, we chose to focus on the change of hospitalization rates in the pre- and post-HACCP period within each division, and it is these changing patterns that we wish to compare and contrast.

The change of hospitalization rates before and after the HACCP was unequal across the divisions. Some divisions did not show expected results: the hospitalization rates in East South Central, West South Central, and South Atlantic divisions were originally decreasing before pre-HACCP, however, they became stable in the post-HACCP period (Figure 2). The East South Central, West South Central and South Atlantic divisions persistently displayed a higher hospitalization rate, estimated maximal hospitalization rate, and relative intensity (Table 2), while their rates of change in hospitalization shifted from decreasing in the pre-HACCP period to unchanged in the post-HACCP period.

Despite the above findings, we cannot nullify the potential beneficial effect of the HACCP regulation in the aforementioned divisions, because the data we used are inappropriate for an intervention-control analysis; it is possible that hospitalization rate would have been even higher if no regulation were introduced at all. In addition, referring to Table 2, the predicted rates of most Census divisions did drop; on average, the predicted minimal rates decreased in most of the divisions except the West South Central (p < 0.05), while the predicted maximal rates also decreased significantly (p < 0.05), except in East South Central. Only the West South Central showed a significant decrease of relative intensity (from 4.22 to 3.26, p < 0.05).

To further discern the possible causes of the patterns observed, investigations involving the collection of data on microbial contamination and detailed bacterial identification, such as serotypes, should be adopted. Nevertheless, the stable rates in three divisions provide important information for geographic targeting.

### Why are the divisions different?

Geographically, the divisions with higher hospitalization rates are mostly situated in the south-eastern United States, where several features might have contributed to these high rates. First, the general health of the elderly in these areas could be relatively worse: in 2000, the all-cause hospitalization rates of elderly aged ≥ 65 in the East South Central and West South Central divisions were 39.7% and 34.4%, respectively, compared to the national average 29.3%. Second, the temperature and humidity in this area tend to be higher [24], thus favouring the growth of thermophiles like *Salmonella*. This argument is further supported by the synchronized and similar temporal patterns of temperature and hospitalizations [15,18,25]. Third, according to Census 1990, sources of drinking water in the area were more likely to be surface water, which is more prone to contamination. Fourth, these divisions also have the highest density of broiler chicken industry in the U.S. [11], possibly leading to increased exposure to *Salmonella *in chicken waste. Fifth, the health-seeking behavior in these mostly rural divisions might also confound with the hospitalization outcome, since distance from the closest hospital was found to influence the rates of hospitalization [26]. Finally, the area is also prone to Atlantic storms and hurricanes, which can cause floods and destroy infrastructures that indirectly lower the quality of the surface water and overwhelm the water treatment systems [27].

### How can seasonality analysis inform health prevention strategies further?

Using the West South Central division as an example, we found that the times to peak were overall similar to the national one, which is about the 25^th ^to 31^st ^week (late June to early August) in a year, with a slight increasing trend throughout the 14 years. The relative intensity is consistently higher compared to the national reference. The combination of high rate, moderate counts (13% of hospitalizations related to *Salmonella *infections were in this division), and consistent time to peak indicates this division to be a potential target for preventive programs against *Salmonella *infections.

Temporally, the annual oscillation peaking at about late June to early August confirmed the thermophile-like behavior of *Salmonella*. However, care must also be taken in attributing any change of risk solely to the physiological property of the pathogen, as human beings also engage in different social activities in various seasons. For instance, increased consumption of unboiled water, outdoor cooking, and accessing surface water for entertainment might have also changed the risk of contracting water- and food-borne diseases.

As time and resources to achieve the Healthy People 2010 goal appear limited, it is important to carry out accurate spatial and temporal targeting for maximum return of benefit. We have identified the South Atlantic, East South Central, and West South Central divisions to be good potential geographic targets, and the 33^rd ^week to be the predicted peak of the rate of hospitalizations due to *Salmonella *infections. Preventive programs, such as water quality monitoring, public service announcement, and other related educational components, can be administered at the most optimal times and places. Of particular concern are the institutionalized elderly, who tend to have longer stays in the hospital and higher mortality rates [28].

### Limitations of this study

There are, however, some limitations to this study. First, we acknowledge that the criteria of coding a disease as *Salmonella *infection could have changed in the study period. However, ICD 003 has been introduced since the Eighth Revision in 1965. In addition, according to National Center for Health Statistics, this code has not been revised or reassigned within the period of our study [29]. We can safely assume that the practice of coding a case of salmonellosis is well established, at least within each division.

Second, there is no validity study on the coding of this particular disease. However, Dubberke et. al. showed a satisfactory agreement between the ICD codes and individual laboratory test results in *Clostridium difficile*, one of the major gastrointestinal infections among the elderly [30]. Studies with similar settings using ICD-9-CM as an outcome of interest were done [31,32]. This analysis provides further insight into applying time series analysis to the national medical records with the purpose of targeting service recipients in both geographic and temporal means. It is also one of the first studies evaluating the potential impact of HACCP implementation on the U.S. elderly.

Fourth, we assumed that the HACCP started at the first week across the nation and the degree of compliance was the same across the division due to the lack of such desirable data. Due to the same reason, we could not study association in different types of meat production facilities. However, chickens were a major transmission route because they have high *Salmonella *prevalence and are preferred by the Americans. According to the USDA, the baseline prevalence rates of testing positive with *Salmonella *in ground chicken and broilers were 45% and 20%, respectively, much higher compared to hog (9%) and beef (3%) [33]. Also, as of 1992, per capita consumption of chicken exceeded that of cattle [34]. We can safely assume that any major improvement brought about by HACCP probably happened through the broiler chicken and ground chicken industries.

Lastly, the CMS dataset, despite its high coverage, is susceptible to under-testing. Also, different health-seeking behaviors might have affected the number of hospitalizations. Up to 92% of all the records in this analysis were "apparent" one-time hospitalizations. Due to the different deidentification processes in each batch of data, there was no valid method to completely identify repeated hospitalizations.

## Conclusion

The trend and seasonality of 14-year worth of hospitalization records related to *Salmonella *infections in the U.S. elderly before and after the implementation of HACCP regulation targeting broiler industries were analyzed. We found that all but two divisions, East South Central and West South Central, demonstrated a significant decrease in hospitalization rates due to *Salmonella *infection (p < 0.05) during the entire study period. When HACCP implementation was considered in the analysis as a potential inflection point, East South Central, West South Central, and South Atlantic showed unexpected results: their rates had been decreasing before HACCP, but then significantly changed to stable after HACCP implementation (p < 0.05 for the three divisions). This phenomenon deserves further study.

We conclude that these three divisions could be potential geographic targets for focused preventions, especially regarding the elderly population. Temporally, harmonic regression indicated that all three divisions had a pronounced seasonal pattern (p < 0.05). We have identified their times to peak to be the 32^nd^-34^th^ weeks of the year, approximately late July to early August. Such information can assist the determination of the timeframe for potential preventive programs.

More research is needed to further isolate other explanatory variables of the seasonality of hospitalizations. Factors such as climate, water quality, dietary intake, demographics, policy, and health-seeking behavior as well as barrier may need to be addressed.

## List of Abbreviations

AK: Alaska state; CDC: Centers for Disease Control and Prevention; CMS: Centers of Medicare and Medicaid Services; FSIS: Food Safety and Inspection Service; HI: Hawaii state; ICD: International Classification of Diseases; ICD-9-CM: International Classification of Diseases, Ninth Revision, Clinical Modification; PR/HACCP: Pathogen Reduction and Hazard Analysis and Critical Control Points Systems; U.S: United States; USDA: United States Department of Agriculture.

## Competing interests

The authors declare that they have no competing interests.

## Authors' contributions

All authors took part in the formulation of the research questions. EN acquired data, devised the analytical plan, and provided statistical guidance. KC developed and revised the analytical plan, carried out the analysis, and composed the manuscript. RR and PW advised on the analysis plan and provided editorial feedbacks. All the authors read and approved the final manuscript.

## Pre-publication history

The pre-publication history for this paper can be accessed here:

http://www.biomedcentral.com/1471-2458/9/447/prepub
